# Visceral leishmaniasis in Iran: Review of the Epidemiological and Clinical Features

**Published:** 2013

**Authors:** Mehdi Mohebali

**Affiliations:** 1 Department of Medical Parasitology and Mycology, School of Public Health, Tehran University of Medical Sciences P. O. Box 14155-6446,Tehran, Iran; 2 Center for Research of Endemic Parasites of Iran (CREPI), Tehran University of Medical Sciences, Tehran, Iran

**Keywords:** Visceral leishmaniasis, Kala-azar, Iran

## Abstract

Visceral leishmaniasis (VL) is a life-threatening vector-borne parasitic disease is distributed in some parts of the new world and old world. The disease is endemic in different parts of Iran. This review article has been focused on major topics of epidemiological aspects and clinical features of VL in Iran for the period of 2002 through 2012. For the detection of VL in humans as well as animal reservoir hosts, anti-*Leishmania* antibodies were detected using direct agglutination test (DAT) as a validated serological test. Parasitological examinations were performed on suspected VL patients as well as canines and rodents. Different molecular methods were used for identification of species and genotype/ or strain of *Leishmania* spp. isolated from infected humans, animal reservoir hosts and vectors. Altogether, 1698 out of 36081 (4.7%) human serum samples collected from 5 distinct geographical zones showed anti-*Leishmania* antibodies at titers ≥ 1:3200 using DAT. The majority of VL cases in the endemic areas were found among children up to 12 years old. Almost 75% of DAT-positive cases (≥1:3200) in endemic areas showed clinical signs and symptoms. Predominant signs and symptoms in 217 hospitalized patients with DAT positive (≥1:3200) results included paleness (99.5%), fever (96.9%), splenomegaly (91.5%), hepatomegaly (53.6%) and lymphadenopathy (21.1%). Integrated VL surveillance system in primary care using DAT, could decrease mortality and morbidity of the disease in the VL endemic areas of the northwestern Iran. Out of 7204 serum samples collected from domestic dogs in various geographical locations of Iran, 879 (12.2%) were DAT sero-positive at titers ≥ 1:320. *L. infantum* as the principal causative agent of the disease was isolated from infected humans, domestic and wild canines and rodents. The principal animal reservoir hosts of the infection are domestic and wild canines. *Ph. kandelakii*, *Ph. perfiliewi transcaucasicus*, *Ph. tobbi* in northwestern Iran; *Ph. major* s.l. (*=Ph. neglectus*)*, Ph. keshishiani*, and *Ph. alexandri* in southern parts of Iran were molecularly and/or parasitologically positive for *L. infantum* infections. The zoonotic form of VL (ZVL) caused by *L. infantum* occurs sporadically in all geographical zones of Iran but in northwestern and southern parts of the country the disease is endemic. DAT as an appropriate and potential tool has been used for sero-diagnosis and sero-epidemiological of VL among humans as well as domestic and wild canines.

## Introduction

Visceral leishmaniasis(VL) also known as kala-azar caused by various *Leishmania* species is a systemic parasitic disease transmitted by female sandflies (1). It is found throughout parts of the old and new worlds and can infect humans as well as domestic and wild animals (1). Domestic dogs (*Canis familiaris*) are principal VL reservoir hosts that can carry either *L. infantum*/ *chagasi* (1). These *Leishmania* species are responsible for a wide spectrum of clinical manifestations in humans, particularly in children up to 12 years old and also immunocompromised adult patients (2). Unlike cutaneous leishmaniasis, which accounts for almost 20,000 new cases per year (3), VL has been reported sporadically in Iran, but the disease is endemic in northwestern and southern areas of the country (4–6) with about 100–300 new cases of VL reported annually.

In the last decade, DAT has been applied vastly for seroepidemiological studies of VL in human and animal reservoirs in various parts of Iran particularly in the endemic areas (4–6). DAT has been also compared to ELISA using intact or soluble antigens of *L. infantum* and indirect fluorescent antibody test (IFAT) for the diagnosis of VL with satisfactory results (7). Since DAT is a simple, cost-effective and field applicable test thus, it has been recommended for seroepidemiological studies as well as early and accurate diagnosis of VL, especially in endemic areas of Iran (1).

This review article was conducted to describe the epidemiological characteristics and clinical features of VL in Iran for the period of 2002-2012.

## Materials and Methods

As VL diagnosis and treatment delay leads to high mortality, an active serological surveillance using the direct agglutination test (DAT) has been established through cooperation of the Provincial Health Services in the endemic VL foci in Iran since last decade (6). Investigations were conducted in northwestern, northeastern, southern, and southeastern Iran, where human VL is endemic in some areas. The criteria for VL endemic areas were based on annual reports documenting the disease in native people, especially in children. The sampling collection was performed either passively or actively.

For passive survey, blood was collected by trained healthcare workers (named *Behvarz*) from all suspected VL patients, who had reported at least three clinical signs, including abdominal distension, paleness, and fever for at least two-week duration. For active survey, a VL serosurvey was carried out in areas where at least three VL cases were confirmed during the previous three years. Children were approached either by a home visit, preschools, or primary schools, as infantile VL occurs more often in the endemic areas of Iran.

Dog populations in villages were selected by non-probable sampling and occasionally multi-stage cluster sampling. Wild canines and rodents were shot and captured around the areas where cases of human VL had been identified previously. Field laboratories equipped with direct agglutination test (DAT) were set up at each location for the purpose of providing a base where patients, especially children up to 12 years of age, and suspected animals could be examined. In these studies, serological tests (particularly DAT), parasitological examinations including direct microscopy and culture, occasionally animal inoculation and molecular characterization techniques, were used.

For DAT, blood samples were prepared from humans and animal reservoir hosts using the following procedures. For humans, blood samples were collected by a finger prick (two drops at 30 µl per drop and were spotted on filter paper (Whatman no.4), allowed to dry, and stored at -20 °C until tested. Alternatively, 100 µl of finger-prick blood samples was collected from suspected patients into two heparin-coated microhematocrit tubes and transferred to local laboratories in a cold box at 4°C.The collected blood was centrifuged at 800 *g* for 5–10 min and the sera were separated and tested. For canines, blood samples were collected after physical examinations by veterinary doctors. Blood samples (∼2.5 ml) were prepared by venipuncture, poured into 10 ml tubes, and processed 4–10 h after collection. The collected blood samples were centrifuged at 800 g for 5–10 min, and the sera were separated and stored at 20°C until examined by DAT.

### DAT performance

The DAT antigen used in these studies were prepared in the Parasitology Department at the School of Health, Tehran University of Medical Sciences (6). All samples were assayed by DAT in remote laboratories and confirmed by the Leishmaniasis Laboratory at the School of Public Health, Tehran University of Medical Sciences (4–6). Antibody titers ≥ 1:3200 were considered as positive results in humans and ≥1:320 for canines (8–14).

### Parasitological examination

Parasitological examinations were performed on suspected humans, rodents, domestic and wild canines. Smears were prepared from bone marrow materials of serologically suspected humans and from skin lesions, livers, spleens, and large lymph nodes of the animals. All prepared smears were stained with Giemsa 10% stain solution and examined microscopically for the presence of amastigote forms of *Leishmania* spp. Some serosity materials or biopsy specimens were collected aseptically and cultured using Novy MacNeal and Nicolle medium and other monophasic culture media. The cultures were incubated at 26°C for up to 8 weeks and examined weekly for the presence of promastigotes. For mass production of promastigotes, monophasic culture media, such as RPMI1640, was used.

### Molecular characterization

Some *Leishmania* promastigotes, which were isolated from infected humans, dogs and vectors were analyzed by isoenzyme and molecular techniques including RAPD-PCR (15–16), PCR-RFLP (17), nested or semi-nested-PCR and sequencing with internal transcribed spacer (ITS) (14) or the *Leishmania nagt* gene, encoding N-acetylglucosamine-1-phosphate transferase and also the cathepsin-1 proteases CPB E/F gene (18), Cytochrome b (Cyt b) and nuclear gene Elongation Factor 1-alpha (EF-1α) (19).

### Vector identification

Adult sandflies were collected with sticky papers, CDC light traps, and aspirators in VL endemic areas. Captured sand flies were characterized morphologically and natural *Leishmania* infection was determined by parasitological (microscopy or/and animal inoculation) and molecular techniques and/or using comparative DNA sequences analyses of a fragment of some selective genes (20–24).

## Results

### Agents

Studies carried out over the last decade by isoenzyme analyses and molecular techniques on 30 *Leishmania* spp. isolated from bone marrow materials of infected humans and spleens of 55 infected domestic and wild canines throughout Iran, showed that *L. infantum* Lon 49 was the principal agent of human and canine VL (4–6, 12–17).

*Leishmania tropica* is typically dermatotropic (25–28) confirmed as a causative agent of VL infection in domestic dogs and humans, particularly in two patients with an HIV/leishmaniasis co-infection with generalized and multiple lesions of skin and visceral involvement and also in an immune-suppressed patient from south part of Iran (13, 25–27). Intraspecies diversity was occurred in some of *L. infantum* isolates and three haplotypes were found in the *Leishmania* species using PCR-RFLP with Acc1 digestion enzyme and sequence analyses of the N-Acetyl Glucosamine Transferase (nagt) Gene (Unpublished Data). Referring to longitudinal study was carried out during 2002-2011, *L. infantum* was known as an accidental causative agent of CL as well as PKDL in the VL endemic areas of Iran. Six CL and 2 post kala-azar dermal leishmaniasis (PKDL) cases caused by *L. infantum* were confirmed in the northwestern Iran (29). Previously, *L. infantum* was isolated from CL patients in the country (30).

### Humans

The first case of human VL in Iran was reported by Pouya (1949) in a boy from the Caspian area in northern Iran (31). Since then, 4300 cases of human VL have been diagnosed in at least 113 cities and districts in Iran throughout the end of 1993 (5). Altogether, more than 2000 cases of VL had been diagnosed in 31 Iranian provinces up to 2012. Totally, 44.6% of the VL infection is reported from northwestern Iran. The average annual number of the diagnosed cases of VL in Iran was 0.449 cases/100,000 inhabitants during last decade. The highest incidence rate of VL was 57 cases/100,000 inhabitants from Ardabil province, northwestern Iran. Based on a prospective survey that was carried out by Davies et al. (1999) on 5671 people in northwestern Iran, the average incidence rate of VL infection was found to be 2.8% in all ages equally at risk in 1985 (9).

**Fig. 1:**
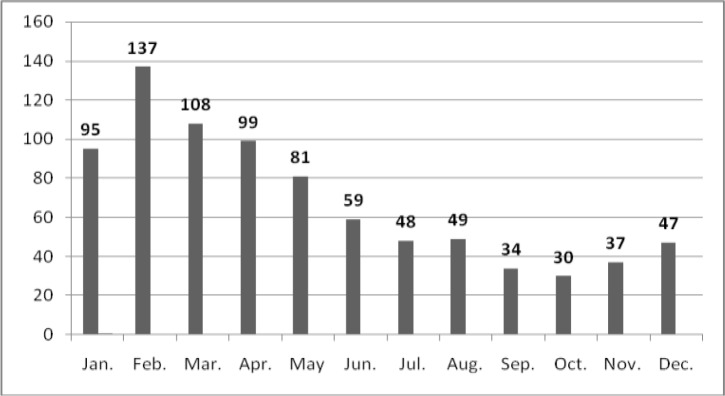
Monthly distribution of 824 diagnosed VL cases detected in last decade (2002-2012)

The DAT was developed and modified as a simple, practical, reliable, and economical technique for the diagnosis and seroepidemiological surveys of human and animal VL in Iran (4–11, 32, 33). A total of 36081 human serum samples (18035 and 18046 samples collected actively and passively, respectively), collected from five distinct geographical zones of Iran from 2002 to 2012. Altogether, 1698 (4.7%) samples (497 in active and 1201 in passive sero surveys) showed anti-*Leishmania* antibodies at a 1:3200 titer or higher (Tables 1, 2), which were considered seropositive results. Almost 75% of DAT-positive cases (≥1:3200) in endemic areas showed clinical signs and symptoms with physical examinations. Predominant signs and symptoms in 217 hospitalized patients with DAT positive (≥1:3200) results included paleness (99.5%), fever (96.9%), splenomegaly (91.5%), hepatomegaly (53.6%) and lymphadenopathy (21.1%). From 246 symptomatic cases of kala-azar, diagnosed in northwestern Iran, 91% were ≤ 5 years-old and only 9% of patients were > 5 years-old. A statistically significant difference was found between males (58%) and females (42%) (P< 0.01). Moreover, 95.0% of the cases lived in rural areas while only 5% lived in urban areas. The annual monthly distribution of VL was determined by examining 824 patients living in northwestern and southern Iran. Five hundred and twenty of cases (63.1%) were diagnosed during cold months (January to May) (Fig. 1).

**Table 1:** Sero-prevalence of human visceral *Leishmania* infection by direct agglutination test (DAT) with anti-*Leishmania infantum* antibodies at ≥1:3200 titers by geographical zones and active case detection (2002 -2012)

Zones	No. of tested	No. of DAT+	sero-prevalence (95% CI)	No. of patients with clinical signs
**North & North-west**	4158	119	2.9 ( 2.3-3.3)	30
**West**	1800	6	0.3 (0.07-0.59)	4
**North-east**	3798	31	0.8(0.53-1.09	5
**Central**	1432	126	8.8 ( 7.25-10.15)	10
**South & South-east**	6847	215	3.1(2.69-3.51)	8
**Total**	18035	497	2.8 (2.47-2.93)	57

**Table 2:** Serological positivity (≥1:3200) in 18035 serum samples tested for human visceral leishmaniasis by direct agglutination test (DAT) in patients referred to remote laboratories by geographical zones (2002-2012)

Zones	No. of tested	No. of DAT^+^	sero-prevalence (95% CI)
**North-west**	12574	638	5.1
**North-east**	562	21	3.7
Central*	4593	396	8.6
South*	317	146	46.1
**Total**	18046	1201	6.6

* Majority of samples were only prepared from hospitalized patients.

This finding associates with second phase of sand fly activities which are initiated from August (34, 35). This distribution showed that the average incubation period of human VL ranged from 2 to 6 months after being bitten by a phlebotomine vector. Global VL mortality is officially estimated at 59,000 (35,000 for males and 24,000 for females) in the world (36), but it is clearly a severe under estimate. In fact there is no point in quoting figures of total mortality due to VL because there is no obligation to report, VL is frequently unrecognized and undiagnosed especially when access to drug is very poor.

There are no current records for the mortality rate of kala-azar in Iran. In a few old reports, the mortality rates of the disease among hospitalized patients varied from 10 to 30% (6, 10). However, based on our retrospective study carried out on 602 hospitalized kala-azar patients in northwestern Iran, only 2.8% of the patients died due to VL (5, 6). Mortality rate was reported 6.2% in children under 1 year old in Fars province, south part of Iran (37). High mortality rates in hospitalized patients were mainly due to the delay in referral to medical centers due to the delay in referral to medical centers.

Based on an intervention study, conducted in northwestern Iran from 2001 to 2007, a VL surveillance system using DAT was established for children aged ≤ 12 years in the primary health system. All cases with clinical VL signs and symptoms and positive DAT results were referred to district hospitals for physical examinations and treatment. During the intervention period, there were only two confirmed VL deaths in the intervention area (including an 8-year-old boy who did not tolerate meglumine antimoniate or amphotericin B regimens for his treatment), while at least 10 VL deaths were registered in the intervention area before the surveillance intervention. Thus, the annual VL mortality rate in children in the intervention area significantly decreased from 0.16 to 0.009/1000 children aged ≤12 years before and after the intervention, respectively (*P* < 0.001) (33), however, 20 VL deaths in children up to 15 years old were registered in the control villages during this period (33). A study was recently conducted on 49 patients suffering from acquired immunodeficiency syndrome (AIDS) in the northeastern Iran. HIV infections were detected by ELISA and confirmed using western blot assays at the AIDS centre of the Khorasan Razavi Province. All collected sera were screened using DAT. The sera with anti-*L. infantum* antibodies at a titre of 1:100 were considered positive for VL infection and serum titration was performed from 1:100 to 1:102400. Nine (18.4%) patients were sero-positive according to DAT. All sero-positive cases showed clinical signs and symptoms. The most predominant signs and symptoms of co-infection of visceral leishmaniasis in HIV-positive patients were pneumonia (n = 2), hepatosplenomegaly (n = 2), lymphadenopathy (n = 2), anaemia (n = 1), prolonged fever (n = 1) and cachexia (n = 1). Our finding shows that visceral leishmaniasis (or kala-azar) is an opportunistic disease in HIV-positive patients in Iran (Unpublished Data).

### Animal hosts

Of 7204 serum samples collected from domestic dogs in villages known as endemic foci of human VL, 879 (12.2%) were seropositive by DAT analysis with titers of ≥1:320 (Table 3). Almost 25% of seropositive dogs showed clinical VL signs (12–14).

**Table 3:** Sero-prevalence of canine visceral *Leishmania* infection by direct agglutination test (DAT) with anti-*Leishmania infantum* antibodies at ≥1:320 titers by geographical zones (2002 -2012)

Zones	No. of tested	No. of DAT^+^	sero-prevalence (95% CI)	*Leishmania* species
**north-west**	3308	608	18.4(16.9-19.6)	*L. infantum L. tropica*
**North-east**	507	40	7.9 (5.4-10.1)	*L. infantum*
**Central**	2525	164	6.5 (5.4-7.3)	*L. infantum L. tropica*
**South-west**	864	67	7.7 (5.9-9.4)	*L. infantum L. tropica*
**Total**	7204	879	12.2(9.6-14.7)	*L. infantum L. tropica*

Parasitological and serological examinations were performed on 39 wild canines (foxes, jackals and wolves). The results showed that 7.7% of the captured wild canines were infected by *L. infantum* Lon 49 (13) (Table 4). A total of 680 rodents (Gerbillidae and Cricetidae) were caught in northwestern Iran. *Leishmania* parasites were detected in livers or spleens of 77 (11.3%) rodents by microscopy examinations (38–40). *Leishmania* spp. were isolated from five *Meriones persicus*, three *Mesocricetus auratus* and three *Cricetulus migratorius* in culture media. Using isoenzyme techniques, the promastigotes isolated from *M. persicus* were characterized as *L. donovani* zymodeme LON 50 and those from *M. auratus* and *C. migratorius* were identified as *L. infantum* (Table 5).

**Table 4:** Results of parasitology and serology tests in wild canines collected in northwestern parts of Iran (2002-2012)

Type of animal	No. of animal	Parasitology	Serology			*Leishmania* species
			DAT	IFA**	ELISA**	
**Foxes**	14	1	1	1	0	*L. infantum*
**Jackals**	13	1	1	1	1	*L. infantum*
Wolves*	12	1	1	1	1	*L. infantum*
						
**Total**	**39**	**3**	**3**	**3**	**2**	*L. infantum*

* *L. infantum* was isolated and identified from an infected wolf in Iran for the first time in 2005.

** Used for confirmation of DAT results

**Table 5:** *Leishmania* species isolates from rodents caught in northwest of Iran during 1998–2004

Rodent species	No. of tested	No. of positive microscopy	No. of positive *Leishmania* species culture	
*Cricetulus migratorius*	23	5	3	*L. infantum*
*Mesocricetus auratus*	4	3	3	*L. infantum*
*Meriones persicus*	469	69	5	*L. infantum* *L. donovani* LON50
*Mus musculus*	124	Nf*	Nf	Nf
*Rattus norvegicus*	60	Nf	Nf	Nf
**Total**	**680**	**77**	**11**	***L. infantum*** ***L. donovani* LON50**

* Not found

*L. infantum* may be transmitted by sandfly from infected rodents to humans in endemic areas. This species of *Leishmania* is zoonotic and has been isolated from humans, domestic dogs, wild canines, and foxes in endemic areas of Iran. In a study carried out in the Semeskandeh area of Mazandaran province in north and Fars province in south parts of Iran, *Leishmania* spp. infection was reported in internal organs of *R. rattus*, but the parasites were not isolated and characterized (41). Feline VL was reported sporadically in some VL endemic areas of Iran (42, 43) but it is not seem the infected cats have the potential role in *L. infantum* transmission cycle in the areas.

### Vectors

Based on epidemiological, parasitological and molecular studies carried out in VL endemic areas of Iran during the last two decades, *Ph.kandelakii* (34, 44), *Ph. perfiliewi transcucasicus* (18–20, 35) and *Ph.tobbi* (44) in northwestern Iran, *Ph. major* s.l., (21, 22), *Ph. keshishiani* (23) and *Ph. alexandri* (24) in southern parts of Iran are recognized as the probable or proved VL vectors. In the recent studies, natural leptomonad infections rates were reported using parasitological and/or molecular examinations in *Ph. perfiliewi* *transcaucasicus* (0.9-1.5%), *Ph. kandelakii* (0.3-1.1%)*, Ph. major* s.l. (3-8.3%), *Ph. keshishiani* (1.1%), *Ph. alexandri* (1.7-4.2%) (18–24, 34, 35, 44–48) and *Ph.tobbi* (25%)^44^. Table 6 shows *Leishmania* infections in *Phlebotomus* spp. in some parts of Iran from 1992 to 2013.

**Table 6:** Proven/probable vectors of VL in Iran by geographical zones, infection rate, *Leishmania* species and method used for *Leishmania* detection (1992-2013)

Zone	Province	District	*Phlebotomus* Spp.	Infection rate(%)	*Leishmania* species	Method of isolation	Investigator(s)
**North-west**	Ardabil	Meshkin-Shahr	*Ph.(Lar.)* *kandelakii*	0.3	*L.infantum*	Parasitology*	Nadim et al.1992
**North-west**	Ardabil	Meshkin-Shahr	*Ph.(Lar.)* *kandelakii*	1.1	*L.infantum*	Nested-PCR	Rassi et al.2005
**North-west**	Ardabil	Germi	*Ph.(Lar.)* *perfiliewi*	0.9	*L.infantum*	Parasitology*	Nadim et al.1992
**North-west**	Ardabil	Germi	*Ph.(Lar.)* *perfiliewi*	1.1	*L.infantum*	PCR	Rassi et al.2009
**North-west**	Ardabil	Bilesavar	*Ph.(Lar.)* *Perfiliewi*	1.5	*L.infantum*	PCR-RFLP	Sanei Dehkordi et al. 2011
**North-west**	Ardabil	Germi	*Ph.(Lar.)* *Perfiliewi*	0.94	*L.infantum/L.donovani*	Semi-nested PCR	Oshaghi et al.2009
**North-west**	East Azerbaijan	Kalibar	*Ph.(Lar.)* *perfiliewi*	2.85	*L.infantum*	Nested-PCR	Parvizi et al.2008
**Nort-west**	East Azerbaijan	Azar-Shahr	*Ph.(Lar.)* *tobbi*	25***	*L.infantum*	PCR-RFLP	Oshaghi et al.2013
**South**	Fars	Ghir-Karzin	*Ph.( Lar.)* *keshishiani*	1.1	*L.infantum*	Parasitology*	Seyedi-Rashti et al.1995
**South**	Fars	Ghir-Karzin	*Ph.( Lar.)* *major* S.l.	3-5	*L.infantum*	Parasitology**	Sahabi et al.1992
**South**	Fars	Ghir-Karzin	*Ph.( Lar.)* *major* S.l.	8.3	*L.infantum*	Nested-PCR	Azizi et al.2008
**South**	Khuzestan		*Ph(Par.)* *alexandri*	1.7	*Leishmania spp*.	Parasitology**	Javadian et al. 1997
**South**	Fars	Nourabad Mamasani	*Ph(Par.)* *alexandri*	4.2	*L.infantum*	Parasitology** Semi-nested PCR	Azizi et al. 2006

* *Leishmania* sp. was inoculated into golden hamsters intraperitoneally and produced VL infection that confirmed by microscopy.

** Natural promastigote infection was found.

*** Of 8 female *Ph. tobbi*, 2 (25%) were found naturally infected with *L. infantum*.

### Brief discussion and conclusion

Infantile VL occurs in various parts of Iran. Ardabil and East Azerbaijan, bordering Armenia and Azerbaijan, are VL endemic areas. Almost 99.0% of VL cases were found among children up to 12 years old in the VL endemic areas. *L. infantum* is the principal agent of human as well as canine VL in Iran. *L. tropica* is the second etiological agent of VL, particularly in immunosuppressed patients. Three haplotypes were recognized in *L. infantum* isolated from domestic dogs. Further studies need to clarify role of the haplotypes in clinical manifestations and geographical distribution of VL. DAT is a practical and reliable serological technique for the sero-diagnosis and sero-epidemiological surveys of clinical suspected VL in human as well as animal reservoir hosts with high validity and reliability.

In some rural areas of Iran, the rate of active kala-azar cases in males was higher than females, but these differences were not statistically significant. It appears that various weather and humidity conditions in the four distinct established geographical zones could influence serological detection of *L. infantum* infections in both humans and dogs.

This is because sand fly activities have been correlated with weather and humidity in northwestern and southern Iran. Increased human *Leishmania* infection has occurred in northwestern Iran where at least 4% of children and 18.4% of domestic dogs showed *L. infantum* infection. It seems that the number of infected dogs from each zone have the greatest potential for disease transmission, due to a large dog population. High canine infection rates should be considered as the most important risk factor of VL in northwestern Iran (6, 12–14, 49). The dog population and canine *L. infantum* infections in northeastern locations were low, thus we found low human seropositivity rates (2.4%). A high seropositivity rate (6.5%) in central Iran can be related to our sampling method because all serum samples were generally prepared from hospitalized patients with clinical VL manifestations. Physicians, especially in areas where *Leishmania* infection is endemic, should be encouraged to test for such infection using DAT and to consider diagnosis of VL more routinely for HIV-infected patients.

Domestic dogs and wild canines are the principal reservoir hosts for *L. infantum* in all four distinct Iranian geographical zones. Determination of the prevalence of canine *Leishmania* infection is necessary to control zoonotic VL in canines, particularly domestic dogs. Wild canines including jackals, foxes and wolves were infected by *L. infantum* and may serve as secondary reservoirs in endemic areas, particularly in villages located in mountainous regions where the transmission sylvatic cycle of VL occurs.

*L. infantum* infection was reported for the first time in a wolf in northwest Iran where human and canine VL are endemic. Also, desert rodents (gerbils) harbor *Leishmania* spp. infection and may, therefore, play a role in transmission of *Leishmania* spp. to humans, particularly children. Further ecological and biological studies of rodents and sand flies are necessary in endemic foci of zoonotic VL of Iran until the exact role of the rodents as animal reservoirs is completely elucidated.

To control VL in Iran, we suggest eliminating stray dogs; identifying suspect leashed dogs by periodic DAT and eliminating those found seropositive; vector control; early /rapid detection of human cases using practical serological tests; treating infected individuals to decrease the disease's mortality rate; and developing public health education.
